# Violence and Agency in Children’s Narratives

**DOI:** 10.1007/s42448-024-00216-w

**Published:** 2024-09-13

**Authors:** Carina Pohl

**Affiliations:** School of Social Work; Institute of Childhood, Youth, and Family, Zurich University of Applied Sciences, Pfingstweidstrasse 96, 8005 Zurich, Switzerland

**Keywords:** Child Maltreatment, Coping Strategy, Response to Violence, Second Story, Physical Abuse, Childhood Studies, Vulnerability, Power Imbalances

## Abstract

The reconstruction of the agency of children who experience family violence is at the centre of this article. The focus is on the subjective experience of agency from the perspective of children, as well as their (in)ability to act and their (lack of) power to act. Based on semi-structured interviews, I analysed how children between the ages of 10 and 14 talk about their experiences of violence and how they present themselves as capable of acting in their narratives - despite their systematically vulnerable position. The analysis shows that establishing spatial distance, offering resistance, and hoping for help are central practices applied by the children. They also develop ‘prevention strategies’ to avoid renewed violence. Children also use the interview situation itself as a moment of empowerment by telling their story in a way that shows how they want to be perceived using their agency. At the same time, however, the limitation of their ability to act is also addressed, which is primarily attributed to their unfavourable position in the power hierarchy with adults. The limitation of their agency is particularly evident in the fact that children cannot stop or prevent violence in the long term despite their strategies. It is thus clearly recognisable that children remain in vulnerable positions despite their agency.

## Introduction

*“But there is also a second story of how the child has responded to these experiences of trauma*,* and this second story is often overlooked. No-one is a passive recipient of trauma. Even children respond in ways to lessen the effects of the trauma […]. This second story is very important.”* (White, 2006, p. 87).

This article focuses on children’s narratives about experiences of violence as a form of traumatic experience. On the one hand, it reconstructs to what extent and in what way children act in violent situations. On the other hand, a special focus is placed on the reconstruction of the agency that children attribute to themselves. Experiences of violence are momentous events in the lives of children, which are accompanied by physical injuries as well as experiences of humiliation and powerlessness (Katz et al., 2021; Kindler, 2023; Strasser, 2013; Sutterlüty, 2004). There are also psychological effects, such as anxiety disorders, depression, posttraumatic stress disorder, posttraumatic guilt, changes in brain structures, and behavioural changes, such as aggression and loss of affect regulation, from which children suffer (Bernard et al., 2014; Briere & Runtz, 1990; Dorresteijn et al., 2019; Korittko, 2020; Lahav et al., 2021; Schulze, 2014b). In addition to research on the effects of violence on children, research has also focused on children’s strategies for action and coping strategies in recent years (Katz et al., 2021; Mullender et al., 2002). Klebanov et al. (2023) were able to identify two strategies through the analysis of forensic interviews with children who have experienced physical violence against themselves: “child’s acceptance of the maltreatment, whereby the child recognizes the abuse is going to happen and there is no way to prevent it” and the attempt to calm the perpetrators, both through an adaptation of their own behaviour, but also by offering them “objects used to abuse” (p. 6). Other findings are from research on domestic violence (DV). Schulze (2014a), for example, conducted narrative interviews with children in Germany, who were in a women’s refuge with their mothers and experienced violence against their mothers. Schulze (2014a) distinguishes between internal effects on the child (feelings of fear, guilt, shame, and sadness) and the children’s actions during the violent situation. These included strategies such as retreating into their room, hiding, trying to protect their mother, and behaving inconspicuously (Schulze, 2014a). Psychologists Georgsson et al. (2011) also researched the experience of violence in partner relationships by interviewing children aged 8 to 14 in Sweden. The children’s behavioural strategies in violent situations included “shying away, interrupting, watching, and obstructed from participating” (Georgsson et al., 2011, p. 120). Kindler (2023) summarizes, that children in situations of neglect, maltreatment or abuse experience feelings of sadness, fear, despair, but also freezing and crying. He further describes: " Many children also describe attempts to distance themselves inwardly from what is happening and to distract or calm themselves, while some try to intervene to mediate or protect, or at least to comfort or help afterwards” (Kindler, 2013, p. 27).

Överlien and Hydén (2009), who also researched DV, linked the children’s action strategies with the agency concept and thus emphasised the position of the child as an actor in violent situations. In this way, children are recognised as victims of violence, but at the same time, their capacity to act is also considered.

The common denominator of the above-mentioned findings is that they explore children’s actions and feelings during DV. Considering poly-victimisation (Finkelhor et al., 2007), it can be assumed that children experience more than one form of violence. So far, narratives about children’s agency during physical violence directed against themselves remain largely unexplored. These “second stories” (White, 2006) are particular important for children because it positions them as agents in this story of trauma and violence, which is most of the time the focus of adult narratives. The fact that too little importance is attached to children’s statements about their actions is based on the difficult access to affected children (Katz et al., 2021). In addition, the affected children are often victimised in the discourse on violence and largely treated as powerless, which emphasizes their vulnerability. As children are seen as capable of acting despite being in highly vulnerable positions (Andresen et al., 2015; Baader, 2015; Klocker, 2007), it can be reasonably assumed that they are also capable of acting in situations of physical violence. The article therefore poses the question: To what extent and in what way do children establish agency in situations of violence that structurally provide them with limited opportunities to act? To answer this question, it is first necessary to develop an understanding of violence and agency.

### Physical Violence

There are various definitions of physical violence in the literature, which differ in the level of detail of the acts of violence and in whether witnessing violence and potential violence are also included. In this article, physical violence is defined as follows: “Physical violence is conceived as an attack on health and life or attacks on the physical or mental integrity of a person, such as beatings or other violent acts such as burning, choking, or shaking. This also includes physical violence as an “educational measure” (author’s note)” (Schweizerische Eidgenossenschaft, 2012, p. 12). Witnessing domestic violence, e.g. violence in a partnership or among siblings, is also taken into account in this article as a form of violence experience from children (ibid.). Based on the WHO (1996), implied and therefore potential acts and threats of violence are also regarded as violence (World Health Organization, 2002). This definition makes it possible to consider both immediate and potential situations of violence. In Switzerland, 30,000–50,000 new cases of child endangerment are recorded every year (Optimus-Studie, 2018). Of all these cases, 20.2 per cent were related to physical violence, mostly in a family context (Optimus-Studie, 2018).

### Children as Actors with Agency

Against this background, the following argument considers children who experience violence as actors capable of taking action despite their vulnerable position. With the concept of children as actors, which assumes that children are experts on the world in which they live (Betz & Eßer, 2016; Nentwig-Gesemann & Thole, 2023), this article focuses on children’s perspectives. On the one hand, they are seen as experts of their living environment, but also as “subjects with diverse experiences, thoughts, feelings, actions, and activities in their everyday lives” (Schulze & Zimmermann, 2012, p. 25) and thus recognised as actors with the capacity to act. In addition, “strengths, resources and also forms of resistance that children and young people use to protect themselves” (Schulze, 2014a, p. 9) become visible and are taken seriously, if children’s perspectives are focused on. In the following argumentation, agency theories initially serve as a “sensitizing concept” (Blumer, 1954) that provides a clear focus and a theoretical framework while maintaining the openness to analyze the nature of agency in a multifaceted way. The theoretical framing of the agency concept opens “a perspective for empirical research that is interested in differentiated analyses of the extent of stubbornness, creativity, and reflexivity that is granted and expected of individuals in respective social contexts” (Scherr, 2012, p. 118). In the following, agency is therefore understood as a relational concept (cf. also Scherr (2012); Eßer (2014). This means that agency is created and made possible or impossible in social constellations (Scherr, 2012; Löwenstein, 2022). This approach offers the opportunity to recognise children not dichotomously, but both as actors and as vulnerable beings, and to focus on their capacity to act under certain conditions (here: family violence and generational order) (cf. Scherr, 2012). This is also achieved by taking into account the quality of agency and recognising that “human individuals are always capable of acting” (Scherr, 2012, p. 113). However, as Scherr (2012) continues, the social constellations and conditions determine the extent of agency. Klocker (2007) describes children’s ability to act in “highly restrictive contexts”, like cases of violence, as ”thin agency” (p. 85), in comparison to “thick agency”, in which many options for action are possible for children. Thus, children can act in violent contexts, but the extent of their capacity to act and alternative courses of action are shaped by the particular context of their experiences of violence. This understanding of agency makes it clear that children cannot overcome violent contexts through their agency but remain vulnerable despite their limited capacity to act. Furthermore, the temporality of agency is an important characteristic (Emirbayer & Mische, 1998). Past experiences leave an imprint on the present and the future, but at the same time, past experiences can also help to shape the future differently (Scherr, 2012).

In order to reconstruct children’s self-assigned agency, the focus is on their narratives. These also provide an insight into their experiences (Lucius-Hoene, 2012). In line with this, children are neither addressed solely as victims of violence nor is there a focus on violence or its effects in „second stories“ (White, 2006). Instead, children are recognised as social actors even in adverse circumstances. This is crucial because “When helplessness becomes the dominant story in a child’s life, their sense of agency is erased.” (Yuen, 2007, p. 4). Children would thus be regarded as powerless without hope for a better future (Yuen, 2007), which this article aims to avoid.

## Method and Material

In the international research network “children's understandings of well-being (cuwb.org)”, the well-being of children is being researched in 26 countries. A standardised research protocol was developed for this purpose, which can be used to collect both verbal data (i.e. interviews and group discussions) and visual data (i.e. drawings) on important people, places, and experiences (Fattore et al., 2021). This protocol was also used in the research project “Childhood Vulnerability and Children's Understandings of Well-being” in Switzerland but was expanded to include specific questions about vulnerability and safety, among other things (Pohl & Pomey, 2023)^1^. Questions on violence were not explicitly asked.

Particular attention was paid to the life context. 56 children (21 female, 32 male, 3 queers) aged 8 to 14 years who were in contact with child and youth welfare services were interviewed. 33 of the interviewed children live in mixed residential care institutions or institutions for boys only, with group settings up to 10 children per group. 23 children regularly visit institutions of child and youth welfare, such as youth centres. The interviewed children were contacted via personal contacts and through professional organizations. The main interests and the interview procedure were presented to all children in the residential care facilities at an initial introductory meeting with the researchers. Actual participation in the interview was then based on the voluntary decision of the children. Therefore, informed consent was obtained from all children interviewed and their legal guardians, which, in addition to confidentiality and voluntariness, also addressed the possibility of terminating the interview at any time. It also included consent to audio recordings. Primarily, the children were able to choose the interview’s location and most of them took place in the welfare institutions.

The data collected was transcribed, anonymised, and analysed based on the grounded theory method (Strauss & Corbin, 1996). For closer examination, the children’s agency was reconstructed using text analysis (based on Helfferich, 2012 and Lucius-Hoene, 2012). Accordingly, the interviewees’ agency becomes apparent on three levels: the narrative sentences, the interaction with interviewers, and their own coping strategies/identity formation during the interview (Lucius-Hoene, 2012). At the level of the narrative sentences, the choice of words, in particular the predicate expressions and semantic roles (Helfferich, 2012; Lucius-Hoene, 2012), is of particular importance. It is also analysed which “movens” (Lucius-Hoene, 2012) are effective, e.g. anonymous powers (“one then has…”), non-human agents (“the alcohol flowed”) or persons (Helfferich, 2012). This also reveals to whom the interviewees attribute responsibility for the event. The responsibility for the event is also linguistically clarified via grammatical modes that mark whether a person sees themselves as active (I have) or as suffering (I became) (Helfferich, 2012; Lucius Hoene 2012). However, the level of interaction with the audience is also analysed, for example through the use of belittling verbs or generalisations. The extent to which the interview is used by the children as an opportunity for empowerment and the extent to which it serves to emotionally cope with the experience of violence is also analysed (Lucius Hoene 2012).

The guiding questions in the analysis were: What happens in the sequence? Who/what causes it and how do the children position themselves in the events? This approach places the children’s narratives at the centre. In their narratives, the children represent themselves such as they “want to be understood in the current situation with regard to agency” (Lucius-Hoene, p. 46). Furthermore, the children can assign themselves a role and thus also define the responsibility for their actions (Lucius-Hoene, 2012). The children’s perspective can also differ from the perspective of adults and their considerations/assumptions about the ability to act in violent situations (Lucius-Hoene, 2012, p. 40). The subjective experience of agency, its (in)ability to act, and its (lack of) power to act, will now be reconstructed from the children’s perspective.

At the centre of this reconstruction are four interviews with children living in residential child and youth care institutions. These four children reported in detail situations of physical violence that were directed against them in their families. Some of the violent situations described took place after the children had been placed in residential care, i.e. at a time when they only went home at weekends and otherwise lived in the children’s homes.

**Table 1:** Selected sequences

Name	Age	Gender	Perpetrator	Point of time	Place
Adam^a^	10	m	Father	Before out-of-home placement	At home
Josie	13	f	Father	Before and during out-of-home placement	At home and in public
Sam	13	d	Mother	Before and during out-of-home placement	At home
Inés	14	f	Father	Before out-of-home placement	At home

^a^ All names are anonymised.

## Results - Children’s Behaviour in Situations of Immediate and Potential Violence

Based on the four selected interviews (see Table 1), the following section firstly traces how children act in immediate violent situations, secondly how they act in potentially violent situations and what prevention strategies they develop, and thirdly to what extent these actions can be understood as a form of agency. Agency is reconstructed with text analysis and with a particular focus on the agency the children attribute themselves.

### Escape: Creating Spatial Separation

Children react to violence in the family for instance by retreating to their room or escaping to a hiding place. Adam, Josie, and Sam, who experience physical violence from a parent, report this behaviour in their interviews. All three children try to escape the immediate violent situation and thus create a spatial separation between themselves and the attacking parent. In doing so, they choose different places to flee to and try to create safe places where they are protected from physical violence and also emotionally feel safe.

Adam decides in favour of his bedroom as a place of escape and describes the situation as follows:

*I: And when he [the father] got angry*,* that wasn’t good*,* did you say?*

*A: No. Then it’s best to run into the room and hope that you [“man” in German] haven’t put the key somewhere where you can’t find it. (Adam*,* lines 375–377)*

In this situation, Adam distances himself from the violent situation by using the impersonal pronoun “you”. The “you” is described as actively acting and being responsible for this action as it “runs” and “hopes”. It is in the hand of the “you” whether the place of protection is reached or not. The verb “hope” emphasises both the dependency and the condition for a successful action strategy to escape violence. For the time being, it remains a “hope” and therefore uncertain whether the chosen strategy will be successful. The phrase “it’s best to run” narrows down the best option within the precarious field of action: running into the bedroom and locking oneself in. At the same time, a recommendation for action is made.

When asked by the interviewer whether he had locked himself in his bedroom, Adam switches to the active form (personal pronoun “I”) and answers the question in the affirmative. He explains: *“Most of the time I didn’t even make it to my room. He [author’s note: his father] is fast*,* he’s fast” (Adam*,* line 621).*

In this situation, Adam uses an active verb form that identifies him as an actor. He therefore also sees himself as responsible for what happens, in this case reaching his bedroom. However, he does not always succeed in doing this, i.e. he acts, but the action appears to be ineffective. An alternative course of action is not mentioned. The extenuating meaning of “most” makes it clear that he seems to have succeeded in reaching his own room a few times. While Adam presents himself as capable of action and active in the first sentence, he now explains the limits of his ability to act with the speed (and thus the physical superiority) of his father, who thus becomes the new actor in the plot. By repeating the same wording, the meaning of the statement (“he is fast”) is emphasised. In this interview extract, he simply addresses his father as “he” without referring to him as his father. Adam thus detaches himself from the relationship constellation “father”-“son”. This is striking insofar as he refers to him as “dad” in other sequences in which he talks about positive experiences. Here, he seems to make a distinction between the perpetrator “he” and his father.

Later, Adam describes his feelings during the violence as follows: “*It hurts. And you think/you think you’re almost a bit abandoned too. By your parents when they beat you.” (Adam*,* lines 556–558)*.

The use of the simile “like” and the mitigating words “almost” and “a bit” soften the feeling of abandonment. He also distances himself from the experience of feeling “abandoned” by rendering the feeling with the verb “think” and the impersonal pronoun “you”. The impression arises that Adam finds it difficult to verbalise his emotional experience during the violence in retrospect, which is why it becomes necessary to fall back on comparisons and cognitions thus distancing himself. In this sequence, the parents are labeled as perpetrators, but not his own, but “your”. This can be interpreted as creating a distance and as a separation from one’s parents and the perpetrators.

Josie also experiences violence at the hands of her father. In addition to her bedroom, the shower is a safe place for her, as she describes in the following sequence:

*I: And in situations where you’re scared*,* what helps you to stop being so scared?*

*J: (…) I just go to my room. Well*,* at the moment I just go to my room and call my boyfriend*,* take my shower things*,* take a shower and while I’m showering*,* I talk to him on the phone. I know*,* sick*,* but I’m like that. Um (…) and then (…) I just talk to him and tell him everything. (Josie*,* lines 484–491)*

Josie deliberately chooses the bathroom because it can be locked. She also remembers to take her mobile phone with her so that she can talk to her boyfriend on the phone. By retreating to the bathroom, she creates a safe place where she decides who is absent (her father) and who is present (her boyfriend). Josie not only makes herself safe physically but also emotionally. By retreating to the bathroom, she creates a safe place for herself, though she knows that it is unusual (*“I know*,* sick*,* but I’m like that”*). She is alone, takes a shower, and makes phone calls, i.e. she activates social resources and thus also creates emotional stability for herself. Violence in the family is often a taboo and the perpetrator often wants to keep it a secret. Josie breaks this taboo by telling her boyfriend about the violence. The strikingly frequent use of first-person words, such as personal pronouns (I) and possessive pronouns (“my boyfriend”, “my shower things”), clearly positions Josie as the acting person, which also puts her in a situation that she can control. It also sounds routine when she describes a list and a sequence of things that she does in such a situation. This shows, on the one hand, that she has probably gone through this sequence many times before and, on the other hand, that she manages to regain control through the sequence and her active behaviour. Her boyfriend plays an important but passive role of listening and being there for her, and at the same time, the friendship provides a protective framework for Josie.

Sam experiences violence from her mother. Unlike Adam and Josie, who seek shelter in the flat, Sam leaves the flat. She runs outside and “sit down somewhere” (line 229).

The three situations described reveal various elements of agency that children attribute to themselves in violent situations. Firstly, in the decision to flee and in particular in the choice of escape location (bedroom, shower, outside). This appears to be a conscious decision on the part of the children (see Adam). Agency is also evident in the decision as to who is allowed to be present/absent at the escape locations. Agency is therefore, on the one hand, withdrawal, but also the activation of one’s own resources, such as the social network, in which a way of processing the violence becomes visible and emotional stability can be regained (see Josie). In addition to creating physical safety by fleeing, it is also about creating emotional safety. The absence of the perpetrators at the escape locations makes the spatial distancing from them visible. This is also reflected in the language, in which the perpetrators remain nameless and are thus distanced from responsibility for the offence or named and thus held accountable.

At this point, however, the limitation of the ability to act is also addressed, namely that the places of escape cannot always be reached. Adam emphasises this, for example, through his father’s physical superiority.

### Resistance: Challenging Power Dynamics

While Josie reacts to violence within the four walls of her home by escaping into the shower, her behavioural strategy changes when she is attacked by her father in a public space. According to a legal decision he is not allowed to see her. In this situation, Josie resists in several ways: on the one hand, she reacts physically and, on the other, cognitively.

*J: Then there’s my father on the other side. He has no right to see me just like that. Without permission. And he can’t just turn up either way. Then he stands there and shouts at me and tells me: ‘You’re coming with me now.’ And I’m like: ‘No*,* ok*,* yes no. I’m not coming with you. I’m going home to my mum now*,* where I belong’. And afterwards he just shouted*,* shouted*,* shouted at me the whole time. Afterwards I said: ‘Leave me alone*,* I’m going home to my mum now because that’s where I belong’. Of course*,* he didn’t realise*,* he grabbed me*,* so he hurt me. He grabbed me*,* picked me up*,* and held me there and there [points to the places on her body]*,* pulled me backwards like that. I got a completely broken back. I have bruises here and there [points to her body]. (Josie*,* lines 552–564)*

On a physical level, Josie tries to defend herself against her father by trying to break away. However, due to her physical inferiority, she is unable to do so. Josie then uses another form of resistance by pointing out her rights and the father’s lack of access rights (“He has no right to see me just like that. Without permission. And he can’t just turn up.“). Even in this directly violent situation, Josie positions herself as an agent by using the action predicates. She is exposed to her father’s actions, who is “on the other side”, as he claims the right to see Josie without permission. She knows her rights but cannot escape her father and his gaze since he is in power during the sequence. What she can do, however, is resist by clearly expressing verbally which behaviour is appropriate and inappropriate and showing her father the limits. It is striking that Josie narrates her part of the speech in detail and merely mentions that her father is shouting without content (“he was shouting”) and is also physically passive (“he was standing there”). Thus, Josie expresses the importance of her words and uses the reference to her rights as a means of strengthening her position in the discussion, because in this way she locates herself in a collective and not only reflects her opinion but also generally valid laws. With the change of situation from a verbal confrontation to a physical attack by the father, the effective power also changes again. The father is the agent and the violence happens to Josie. The active verb form changes into a passive one (“He grabbed me, so he hurt me”, “held me”, “pulled me back”). She suffers the violence and is in physical pain.

In this situation, Josie refers to her father as “my father”, but he is on the “other side”, which creates a spatial distance. The distancing and emphasis on the spatial separation from her father is also visible in the statement, “I’m going home to my mum now because that’s where I belong” (line 431). In addition to distancing herself from her father, Josie creates a sense of belonging to her mother.

Josie’s resistance, her insistence on her rights in a dangerous situation, and confronting her father reflect her agency. Josie’s agency is also evident in the fact that she creates new power structures by establishing affiliations (here: to the legal framework and to her mother). In doing so, she opens two fronts and positions herself with a collective/her mother against her father. Josie also uses the interview situation as a moment of empowerment in which agency becomes visible by reproducing her language in detail and depriving her father of the right to speak, breaking it down to a meaningless “shouting”. Josie can clearly categorise the violent situation with her father as wrong because she knows that her father has no right of access. This knowledge of her rights helps her to assess situations and gives her the self-confidence to act. She recognises that she has the right to defend herself and get help. Josie cannot compensate for her physical inferiority, but on a cognitive level, she can put up resistance.

### Outreach: Hoping and Waiting for Others to help

It is known from research on violence that it is a major hurdle for young people to actively seek help and articulate violence. From the children’s perspective, Seith (2013) cites, for example, shame, and conflicts of loyalty, but also the children’s fear that they will not be believed as reasons for that. Sam and Josie use talking to adults as a strategy and hope that they will help them. They use this strategy in addition to escaping (Sam and Josie) and resistance (Josie). Inés also hopes for the help and intervention of others but cannot/does not want to articulate this. Sam’s and Inés’s hope for help remains unfulfilled, while Josie receives the help she had hoped for.

When Sam runs away from her mother’s violence, she meets her father there. He lives apart from his family. Sam confides in her father and hopes for help.

*S: And then my father came*,* came*,* yes*,* exactly. To visit us. And afterwards he saw me and asked me what had happened. And I told him afterwards what had happened. (Sam*,* lines 229–231)*

The active agent in this sequence is initially Sam’s father, to whom the ability to act is attributed via the action predicates. The father comes, sees Sam and her situation. Sam remains passive. She is seen and spoken to by her father. Only then does Sam become active and tells her father about the violent experience.

His reaction seems to disappoint Sam:

*S: And the only thing he said was: ‘Yes*,* your mum is a retarded [“behindert” as a curse word in German] woman.’. And the thing is*,* my father is not the kind of person who*,* for example*,* if someone were to bully me now*,* he couldn’t go to them and say: ‘Hey*,* leave my daughter alone’ or something like that. He can’t do that. He can’t get angry and tell me like that/ he can’t/like protect you could say or defend or something like that*,* he can’t do that. And it never went against my mum either*,* that’s why it always went on like that*,* that my mum hit me for the stupidest things. (Sam*,* lines 231–237)*

Although the father agrees with Sam and verbally opposes her mother (“your mother is a retarded woman”), it becomes clear that Sam would have liked him to react differently and more strongly, namely to intervene, stand up for her, and protect her. Linguistically, also an ambivalence becomes visible. On the one hand, Sam releases her father from responsibility for his inaction by emphasising that he is not capable of intervening. On the other hand, she establishes a causal link between the father’s inaction and the continued experience of violence. The father is therefore in a powerful position. Sam remains passive in this sequence linguistically. She experiences potential bullying and she does not position herself in a powerful position, but hopes for her father’s support.

Sam’s agency is conveyed when she talks about the violence and when she confides in her father. The limits of her agency become apparent as she cannot confront her mother and her violence on her own but must hope for and depend on help.

Inès shows a different kind of reaction to violence. She also experiences violence at the hands of her father. Unlike Sam, however, Inés does not seem to have somebody she could confide in. Instead, she generally states that children need protection from adults and that they should look out for them.

*I: They [author’s note: adults] know that their (…) children are being abused next door*,* for example*,* and they don’t say anything. You must say something about it. (Inés*,* lines*,* 1017–1018).*

In this sequence, the people are not named in detail at first. However, it becomes clear that adults are aware of the violence against children but say nothing about it. Inés addresses an unnamed collective by using the impersonal pronoun “you”. From her perspective, however, it is not an option but a “must” that something is said in the event of abuse.When asked by the interviewer whether Inés herself had witnessed somebody looking the other way when faced with violence, Inés refers to her neighbour.

*I: Yes*,* she knew and she didn’t say anything. That we needed help… (Inés*,* line 1037)*.

Linguistically, the neighbour is marked as capable of acting and therefore responsible for acting or not acting. This is not just about creating safety for Inés, but also for her twin brother (“we”). As with Sam, Inés’ desire for protection and recognition of her emergency situation is also clear.

From the outside, Inés’ actions during violent situations are not visible. And yet she positions herself in the interview as active by sharing her thoughts and wishes. In her head, she is cognitively active and acting. Inés agency is visible in the fact, that she does not give up and resign, but keeps hoping. Perhaps hoping is the only possibility for action that Inés can show in violent situations because she is denied alternative options.

In contrast to Sam and Inés, Josie gets the help she hopes for. In the sequence described above, Josie is attacked in a public space and reacts with resistance (see Chap. 3.2.), but also by shouting for help. In response to Josie’s screams, a policeman who happens to be driving by, recognises the dangerous situation. He asks the father to let go of Josie, but when he does not comply, the policeman pushes the father away and frees Josie from his’s grip (lines 583 ff.). Apart from the screaming, Josie positioned herself as passive in the sequence. The agents are the father and the policeman.

Agency can be seen here in the fact that children can turn to adults for help. At the same time, children are dependent on adults to act. With (Bryant & Ellard, 2015), hope can also be seen as an action and agency. In this case agency is to be able to hope in a bad situation and not give up.

### Preventive Action Strategies

In addition to these three forms of action during violent situations, there are also prevention strategies against new potential violence showing that children are able to recognise danger in the family and identify dangerous patterns of violence. This is possible when children have already experienced violent situations and are sensitive to their parents’ moods and behaviour and can assess when violence may happen. Accordingly, children adapt their behaviour in the form of a so-called “projection” (Scherr, 2012, see Chap. 1).

Adam is particularly apt in using these prevention strategies. He says that when his father gets angry, *“then he is really angry. That’s what I mean now*,* it’s really better if you leave him alone. And walk away a bit”* (lines 604–606). His prevention strategy is initially not to upset his father. This shows that he can conclude how his behaviour affects his father’s violent outbursts. In this way, he takes some responsibility for his father’s outbursts and realises that his own actions have consequences. Another prevention strategy is to leave the potentially dangerous situation. A third strategy is to take precautions for a successful escape, e.g. to have the key at hand (see Chap. 3.1.).

In this sequence, the father remains a nameless “he” and the power to act is usually attributed to an unknown object via the impersonal pronoun “you”. Adam does not elaborate on whether the strategies of remaining the father calm (“leave him alone”) and escaping (“walk away a bit”) are successful.

Another preventative measure concerns his behaviour in the future. Adam distances himself from violence and emphasises that he wants to provide for his own future family. When asked what caring signifies for him, he says:

A: *I don’t think I would be such a strict father with my child. I would play with him a lot and stuff*,* because I would NEVER hit him. Never. Because I know what that feels like and I don’t want it to go on for generations. (Adam*,* lines 548–552)*

In this sequence, Adam appears as an active person and thus emphasises that he is taking his future into his own hands. At the same time, he distances himself from his father and from his family (“not going on for generations”). He thus takes responsibility for his actions. As being beaten is associated with pain and abandonment for him (see Chap. 3.1.), it is clear that he not only wants to create a non-violent but also an emotionally safe environment for his children.

These prevention strategies also show new aspects of agency in the sense of projection relating to strategy development in the present, shaping the future, and decisions about the future. Furthermore, agency becomes apparent through the conscious dissociation of violent behaviour. However, it is also clear how fragile this agency is since it depends on the father’s behaviour.

## Discussion

The empirical material reveals various elements of the introduced concept of agency. There are no children in the sample who just see themselves as a person suffering physical violence, but rather describe their actions despite their vulnerability and limited room for manoeuvre, thus positioning themselves as an actor (cf. Lucius Hoene, 2012). These results can be linked back to the theoretical understanding of agency (Scherr, 2012) and the significance of second stories (White, 2006). The limits of agency also becomes visible.

### The importance of social constellations

In social constellations, children suffer from their parents’ violence and are forced to act as a result. Their agency is reflected in the negotiation process between distance and closeness to their parents. This is particularly visible in the spatial distancing from the parents, but also linguistically through the decoupling of the parents from their role as perpetrators. In addition, the causes of violence are localised in the circumstances to which parents are exposed. In doing so, the children release their parents from responsibility for the violence. At the same time, this could be read as an attempt to maintain the existing social relationship between parent and child. Another attempt in this regard could be the (partial) assumption of responsibility by the children for outbreaks of violence.

In social constellations, children are hurt, whereas in other social constellations, children find support. Children’s agency appears when they leave perpetrator-victim constellations and enter supportive constellations, such as boyfriend-girlfriend or mother-daughter relationships. They create affiliations that seem to give them a sense of security. This changes the balance of power, because the children do not stand alone against the perpetrators, but together with trusted persons/collectives.

### Quality of the agency

The quality of agency is determined by social structures and contexts. (cf. Abebe, 2019; Klocker, 2007). In violent situations, children act within an extremely limited and very narrow sphere of action. Sometimes it is just possible for children to hope and to be active in their thoughts (cf. Bryant & Ellard, 2015; Katz et al., 2020). However, it is crucial to speak of (‘thin’) agency in these situations to recognize the children’s ability to act, but also to acknowledge the injustice these children have experienced and the precarious situation they had to face. Klocker (2007) formulates: “When their agency is identified as ‘thin’, rather than as being non-existent, this enables acknowledgement both of their difficult circumstances and their efforts to survive and to build better lives.” (p. 92). By using the few opportunities to act, the children distance themselves from being turned into objects and instead act as subjects (cf. also Jacquemin, 2004; Klocker, 2007). Thus, their agency, even in violent situations become visible. Children make full use of this room for manoeuvre and even develop several strategies, like escaping, challenging power dynamics, hoping for help, developing prevention strategies, and consider options for action. This enables them to escape the violence depending on the situation.

However, the limits of the agency also become visible. The children have no way of escaping violence in the long term, and they cannot prevent violence from happening again. Despite their agency, they remain exposed to violence. The children explain this with their physical inferiority and their exposure to the moods of adults. In this way, the adults are placed in more powerful positions which the children are exposed to.

### Temporality of agency

Past experiences shape the children’s present and future. However, the children refuse to accept the circle of violence, which is often one result of experienced child maltreatment (Fitton et al., 2020), and decide to shape the future differently (cf. Scherr, 2012; Emirbayer & Mische 1988). The children develop prevention strategies (cf. Asdigian & Finkelhor, 1995; Katz et al., 2021) to break this spiral of violence, which results in a projection. In doing so, they want to create safety for themselves in the present, but also safety for their future children (cf. Bryant & Ellard, 2015) and to break through the transmission of violent behaviour. Temporality is also reflected in the fact that children can escape violence for a given time.

### Narration as empowerment

Against the background of the so-called second stories (cf. White, 2006), it can be deduced that children do not experience violent situations as passive observers, but rather explicitly emphasise their ability to act, especially in retrospection. This sometimes manifests itself in the fact that the children actively seek out contact persons to break the silence regarding the violence. In this way, they strive for support and comfort. According to Lucius-Hoene (2012), the interview can be used as an act of empowerment. This becomes particularly clear in the way the children take ownership of the interview because they see it as a space where they can speak freely. This appeared to give them a sense of empowerment, helping them to interpret and act on their story. In the interviews, the children decide to talk about the violence they have experienced and depict themselves as actors with agency by determining the narrated content and assigning agency and speaking roles. Through the self-chosen forms of speaking about violence and the focal points in the narratives, the children regain control over their story and thus also use the speaking space offered to cope with the violence they have experienced.

The children use the interview framework to address their feelings like being abandoned and fear, to draw attention to injustice, to describe the physical consequences of the violence, and psychological reactions such as trembling with fear and pain. They address their ability to act, but also its limits. Most children portray themselves as active and therefore capable of acting. Through this positioning, the children regain control over what is happening. This positioning is reversed in moments when they are physically inferior to the perpetrators.

In addition, the children try to legitimise their parents’ actions and find reasons for their behaviour. The search for legitimisation and explanation can also be seen as a form of agency according to Lucius-Hoene (2012). This coping strategy is important for identity work since it serves to integrate the experiences into the course of life and helps to endure the tension between the role of parents as perpetrators and caregivers at the same time.

## Conclusion

The aim of this article is to show the extent to which children can show agency in extremely precarious situations, specifically during violence, to understand the actions of children in such situations and to reconstruct their self-attributed agency. A variety of possible courses of action and strategies for action emerged, some of which children use consciously to escape violence in the best possible way and remain capable of acting despite unequal power relations. The children used the interview situation as an act of empowerment by talking about violence and linking it to the topics of violence and agency. In doing so, they do not focus on the acts of violence they experienced, nor do they see themselves as mere victims of violence. On the contrary: children consider themselves as capable of acting in violent situations and want to be perceived as such by others (cf. Lucius-Hoene 2012). However, they also recognize the limits of their ability to act, and their limited effectiveness. The perspective of children, who have experienced violence against themselves and of whom some are still in violent situations, expands the current discourse on violence. They also contribute to a holistic understanding of agency.

Furthermore, the entanglement between vulnerability and agency becomes visible (cf. Andresen et al., 2015; Heite & Magyar-Haas, 2020). This is because children’s agency cannot end the violence in the long term and does not protect them from future violence. They remain exposed to the adults and the unequal power imbalances. The creation of agency is also accompanied by false assumptions of responsibility and blame for the physical assaults, leaving the children in precarious, vulnerable, and less empowered positions. This entanglement must be taken into account when talking to children. Questions about “how did that make you feel”, which are aimed at vulnerability, and “what did you do”, which can produce narratives about agency (Överlien, 2017), should therefore be combined to open questions that do not deny children their vulnerability nor their ability to act, but leave it up to them to set their own priorities in the narratives. Then children are encouraged to tell their “second stories” (White, 2006). This creates a platform to talk about both: the vulnerability and the associated pain and emotions, as well as the agency through which children can regain some control over their story and assume a more powerful position.

## Data Availability

The data that support the findings of this study are not openly available due to reasons of sensitivity and ethical restrictions.

## Footnotes

1 The study is ethically approved by the University of Zurich.
